# Surgical HDU admissions: utilisation, organ support and finance

**DOI:** 10.1186/cc13202

**Published:** 2014-03-17

**Authors:** G Brakmane, A Molokhia

**Affiliations:** 1 University Hospital Lewisham, London, UK

## Introduction

The aim was to explore the HDU booking process, organ support requirements and financial implications as the current approach to booking surgical cases for HDU has somewhat been based on a ‘just in case' principle.

## Methods

The booking and admission details from October 2012 to July 2013 were gathered from the HDU log-book and Ward Watcher software. The cost of HDU beds and staff were provided by the finance department. Data were analysed using MS Excel and SPSS software.

## Results

**Figure 1:**
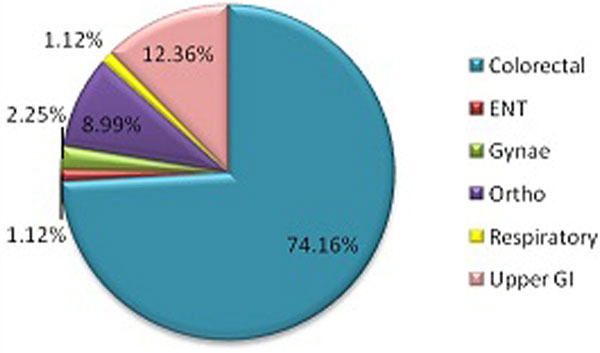
**Admissions per speciality**.

**Figure 2:**
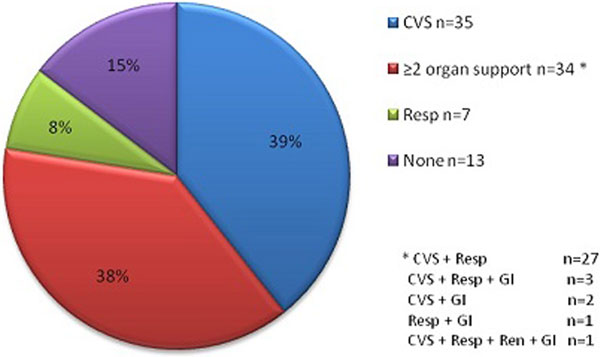
**Organ support requirements**.

There were 105 handwritten bookings over 10 months, several missing essential data. Only 89 admissions actually took place; 61 were elective, 66 colorectal, six urgent, 19 scheduled and three unspecified (Figure 1). Most patients required basic cardiovascular and respiratory support (Figure 2). Data revealed 16 cancellations. Each booking requires allocated nursing staff which bears a basic cost of £23/hour (estimated annual loss >£10.000). If we add the daily cost of basic HDU care (approximately £1,000 for single organ support), the losses rise rapidly. We also registered 38 days in delayed discharges. This was predominantly due to ward bed shortages. In financial terms there is at least £500/day difference between surgical and HDU (potential annual loss >£22,000). In addition there were 13 patients who spent a total of 37 days on the HDU but did not require an HDU level of care.

## Conclusion

The currently limited resource is not being utilised effectively with implications on both staff deployment and finances. A more holistic approach is needed where all requests are reviewed by a consultant anaesthetist in conjunction with preadmission data. This could better identify patients for HDU care and potentially decrease cancellations. We have developed an improved booking form for this purpose.

